# Supplementary material for: N-acetylcysteine (NAC), an anti-oxidant, does not improve bone mechanical properties in a rat model of progressive chronic kidney disease-mineral bone disorder
Source: PLoS One. 2020 Mar 23;15(3):e0230379. doi: 10.1371/journal.pone.0230379 (PMC7089527; doi:10.1371/journal.pone.0230379)
Supplement: Attachment — Submitted filename: Response to Reviewers.docx [file pone.0230379.s005.docx]

**Reviewer #1:**

1) It is well known that the level of systemic or local oxidative stress increases in CKD condition. The previous study of the authors also reported 8-OHdG in serum was increased in 35weeks old Cy/+ rats (Avin KG, et al PLOS ONE 2016). However, why were the levels of oxidative stress marker in serum similar between Normal and CKD rats in this study? The authors should discuss this point.

*The reviewer is certainly correct that we and others have noted elevated oxidative stress in CKD. We in fact have seen altered oxidative stress in two papers using this animal model. We are unclear as to the reasons they were not elevated here, as that was certainly the basis for the study/intervention with NAC. We have tried to make this explicitly clear in the discussion. Unfortunately, we don’t have great hypotheses for this finding (or lack of finding).*

2) Does the degree of redox in bone tissue reflect on the degree of redox in serum? Did the authors determine the level of anti-oxidant markers such as superoxide dismutase, or glutathione in both bone tissue and serum? It is necessary for the authors to present these data or argue this point while using previous studies.

*The reviewer raises a great question about relation of serum and bone levels but unfortunately we are not able to make those assessments in both bone and serum. We have added this as a limitation of the work.*

3) What does the reducing of bone mechanical properties in CKD rats depend on in this study? Does the amount of total bone AGEs, or pentosidine correlate with bone mechanical properties?

*We have added a correlation matrix table and a limited amount of text to the discussion. Total bone AGEs and 8-OHdG are both are negatively correlated to mechanical properties (r values around -0.3 for many variables). These relationships are not overly strong, albeit statistically significant.*

4) What the level of serum creatinine or creatinine clearance in their CKD rats? This data is important to understand CKD related bone abnormalities. The authors should describe this data.

*We did not measure serum or urine creatinine in the current study. We have previously undertaken creatine clearance in our animal model and shown that they correlated to BUN. in our animal model. Thus we are confident that BUN accurately reflects that CKD animals in this study had kidney disease and that NAC did not affect the degree of altered kidney function.*

5) Was the method of total bone AGEs assessed by microplate reader authorized in this case? Did the value obtained by this method reflect AGEs content? The authors should address much more in detail about this method.

*We followed previously published methods for measure of total AGEs. We have added citations to the methods section.*

6) The authors described calcium and phosphorus content in materials and methods. It is well known that protein and fat content in diet affects kidney function and bone health. It is better for readers to have this information.

*We have included dietary values of protein (17.7%) and fat (5.2%) in the methods..*

7) In the discussion, the authors described no effect of NAC on the aorta in CKD animals. Which data was this shown by?

*We have removed reference to the aorta – sorry for the confusion.*

8) In Table 4, was the data of pentosidine content in the two CKD groups correct? Was this data not replaced? It is recommended to check this data.

*We have verified that the pentosidine data in Table 4 is correct.*

Reviewer #2:

1. The format of the tables should be modified to standard three-line table or four-line table and the results of pairwise comparisons should be indicated used the symbols, like “*”or others.

*We are happy to edit the table as needed for publication but are unsure what the reviewer is requesting here.*

2. In the figures, the results of pairwise comparisons should also be indicated used the symbols and horizontal lines, which indicated the difference occurred in indicated two groups.

*We have revised our figures to make it clear that the notations in the upper left corner are main effect and interaction p values. In figures 1B and 2B, where there are main effects, we find it more complicated to add specific notations to the figure – these main effects make it clear that either drug or disease are different. For Figure 3 we have a notation in the figure legend that the * symbol represents difference from all other groups. We hope this is clear.*

3. In the part of “The effect of NAC on total bone AGE accumulation and skeletal collagen cross-linking of femoral cortical bone”, the statement of ‘Assessment of enzymatic collagen crosslinks and pentosidine by HPLC revealed that pyridinoline levels per unit collagen were significantly lower in CKD animals compared to NL with no effect of NAC treatment (Table 4).’ is not clear.

*This statement matches the table, which shows a significant main effect of disease for pyridinoline (0.0001) but not for treatment (and no interaction).*

4. In the ‘Statistics’ part, the statement should be clearer and should tell the readers the meaning of “interaction”.

*We have clarified the results section and the tables.*

5. The authors could make some bivariate correlation analysis, such as the correlation analysis of oxidative stress indicators with the AGEs, or the correlation relationship of AGEs with bone indicators of micro CT and bone biomechanics.

*We have added a correlation matrix table to the paper (Table 5) and referenced it in the results section.*

6. The expression and grammar should be improved, errors about singular and plural should be corrected.

*We have tried to correct grammar where we saw inconsistency.*
